# Inflammatory markers are associated with psychomotor slowing in patients with schizophrenia compared to healthy controls

**DOI:** 10.1038/s41537-020-0098-4

**Published:** 2020-04-01

**Authors:** David R. Goldsmith, Nicholas Massa, Bradley D. Pearce, Evanthia C. Wommack, Alaaeddin Alrohaibani, Neha Goel, Bruce Cuthbert, Molly Fargotstein, Jennifer C. Felger, Ebrahim Haroon, Andrew H. Miller, Erica Duncan

**Affiliations:** 1 Emory University School of Medicine, Department of Psychiatry and Behavioral Sciences, Atlanta, GA USA; 2 Atlanta Veterans Affairs Medical Center, Decatur, GA USA; 3 Rollins School of Public Health, Emory University, Atlanta, GA USA

**Keywords:** Biomarkers, Schizophrenia

## Abstract

Patients with schizophrenia exhibit psychomotor deficits that are associated with poor functional outcomes. One pathway that may be associated with psychomotor slowing is inflammation. Inflammatory markers have been shown to be elevated in patients with schizophrenia and are associated with psychomotor deficits in both animal and human studies. Forty-three patients with schizophrenia and 29 healthy controls were recruited and underwent a battery of psychomotor tasks. The following immune measures in peripheral blood were assayed: IL-6, IL-1 beta, IL-10, TNF, MCP-1, IL-6sr, IL-1RA, and TNFR2. Generalized linear models were used to determine which immune markers, in addition to their interaction with diagnosis, were associated with performance on the psychomotor tasks. As expected, patients with schizophrenia demonstrated slower performance compared with healthy controls on the finger tapping test (FTT, tested on dominant and non-dominant hands), trail making test (TMT), and symbol coding test (SC). Interactive effects with diagnosis were found for TNF, IL-10, IL-6sr, and TNFR2 for the FTT (dominant), IL-10 and IL-6sr for FTT (non-dominant), TNF and IL-10 for TMT and TNF, IL-10, IL-6sr, TNFR2, and IL-1RA for SC. The results of this study provide evidence that peripheral inflammatory markers contribute to psychomotor slowing in patients with schizophrenia. These data are consistent with a growing literature, demonstrating that inflammation may target the basal ganglia to contribute to psychomotor deficits as is seen in other psychiatric disorders such as depression. These data also indicate that psychomotor speed may be a relevant construct to target in studies of the immune system in schizophrenia.

## Introduction

Deficits in psychomotor activity and performance on neurocognitive tasks of psychomotor speed and psychomotor processing speed have been clinically described and reliably demonstrated in patients with schizophrenia^1^. Early descriptions of the disorder^2,3^ recognized psychomotor slowing as a core feature of the illness. More recent work has suggested that it may be an independent cognitive domain that is impaired in schizophrenia^4^. Indeed, speed of processing is included in the “Measurement and Treatment Research to Improve Cognition in Schizophrenia” (MATRICS) Consensus Cognitive Battery (MCCB) that includes tasks of both psychomotor speed (i.e., trail making test: part A) and psychomotor processing speed (i.e., symbol coding test; SC)^5^.

Psychomotor slowing appears to be present at the onset of the disorder: studies have observed slowing in antipsychotic-naive first-episode patients, whose performance do not appear to improve with treatment and may worsen over the course of illness^6^. Psychomotor slowing may also be associated with the deficit syndrome, characterized by primary and enduring negative symptoms^7^. Moreover, deficits in psychomotor activity have been demonstrated in individuals at clinical high risk for psychosis (before conversion to a diagnosable primary psychotic disorder) as well as in first-degree relatives of patients with psychosis, suggesting that there may be a genetic component^8^ and that psychomotor deficits may be an intermediate phenotype for the disorder^9^. Importantly, psychomotor slowing has been associated with worse functional and social outcomes in patients with schizophrenia^10,11^.

Few studies have specifically addressed the neuroanatomical underpinnings of psychomotor slowing in patients with schizophrenia, though basal ganglia circuitry has been consistently implicated^12–15^. These findings are consistent with dysfunction in dopamine-rich regions of the basal ganglia, such as the dorsal striatum as is found in patients with major depressive disorder who also exhibit psychomotor retardation^16,17^.

One pathway that may contribute to psychomotor slowing in patients with schizophrenia is inflammation. Indeed, immune system abnormalities are posited to be involved in the neurodevelopment of schizophrenia^18–24^. Immune dysfunction is seen in patients with schizophrenia as evidenced by meta-analyses, demonstrating alterations in various inflammatory markers, including inflammatory cytokines (both in peripheral blood and cerebrospinal fluid)^25–28^, acute phase reactants such as C-reactive protein (CRP)^29,30^, and chemokines^31^. Moreover, inflammatory markers, including tumor necrosis factor (TNF), are associated with the deficit syndrome, which, as noted above, has been linked to reduced psychomotor speed^32^. Pre-clinical work has also shown that rodents exposed to inflammatory cytokines or inflammatory stimuli (e.g., interleukin (IL)-6 or lipopolysaccharide) exhibit decreased locomotor activity^33,34^. Similarly, a number of human neuroimaging studies have consistently shown that induction of inflammation (via a number of different stimuli, including interferon-alpha, typhoid vaccination, and endotoxin) alters both neural activity and dopamine metabolism in regions of the basal ganglia, including the dorsal striatum^35–38^.

As such, we sought to examine the relationship between immunologic measures including inflammatory markers and performance on a battery of psychomotor tasks in patients with schizophrenia. To investigate whether the hypothesized relationship between inflammatory markers and psychomotor deficits is specific to patients with schizophrenia, we also included a healthy control group. Furthermore, the tasks included in this analysis allow for investigation of a variety of psychomotor tests ranging from those that reflect a purely motor task (e.g., finger tapping) to those that involve more cognitive demand and cortical activity (e.g., trail making test (TMT) and SC). This approach may also allow for further understanding of distinctions between tasks of psychomotor speed and psychomotor processing speed, such as SC, as there is some debate as to whether they may reflect different processes^39–41^.

## Results

### Study sample characteristics

Sociodemographic variables, psychomotor task performance, and immune marker concentrations for the 43 patients with schizophrenia and 29 healthy controls are shown in Table 1.

**Table 1:** Sociodemographic characteristics, estimated marginal means of psychomotor task performance, and immune marker concentrations of patients with schizophrenia and healthy controls.

	Schizophrenia (*N* = 43)	Controls (*N* = 29)		
	*N* (%)	*N* (%)	*χ*^2^	*P* value
Sex (male)	39 (90.7%)	24 (82.8%)	0.998	0.318
Race (black)	41 (95.3%)	23 (79.3%)	4.511	0.034
Smoker	24 (55.8%)	15 (51.7%)	0.117	0.733
	*Mean (SD)*	*Mean (SD)*	*T*	
Age (years)	51.47 (9.35)	53.07 (10.75)	0.672	0.504
BMI	31.60^†^ (5.43)	29.46* (5.84)	−1.520	0.134
PANSS Total	58.37 (11.73)			
	*Mean (SE)*	*Mean (SE)*	*Wald* χ^*2*^	
FTT (dom)	258.66 (13.77)	308.23 (11.25)	11.085	0.001
FTT (non-dom)	226.75 (10.91)	269.18 (10.88)	11.867	0.001
TMT	40.57 (3.44)	28.36 (1.71)	15.980	<0.001
SC	44.02 (2.51)	52.74 (2.19)	10.622	0.001
RTT	330.72^‡^ (28.98)	312.79 (20.66)	0.798	0.372
TNF (pg/ml)	4.23 (0.36)	3.61 (0.22)	3.428	0.064
IL-1b (pg/ml)	0.58 (0.19)	0.61 (0.02)	1.478	0.224
IL-6 (pg/ml)	3.56 (0.31)	3.48** (0.22)	0.075	0.784
IL-10 (pg/ml)	0.48^‡^ (0.04)	0.44 (0.03)	0.713	0.398
MCP-1 (pg/ml)	167.98^†^ (3.69)	163.75** (2.87)	1.081	0.298
IL-1RA (ng/ml)	539.46^†^ (70.30)	400.20** (40.89)	5.134	0.023
IL-6sr (ng/ml)	41678.63^§^ (2382.86)	41666.51** (2068.03)	0.000	0.996
TNFR2 (ng/ml)	2616.95^†^ (388.57)	2420.45** (306.76)	0.413	0.520

Means and percentages of demographic variables between groups compared with *t* tests and chi-square tests, respectively; *SD* standard deviation, *SE* standard error, *BMI* body mass index, *PANSS* positive and negative syndrome scale, *FTT (dom)* finger tapping task (dominant hand; number of taps), *FTT (non-dom)* finger tapping task (non-dominant hand; number of taps), *TMT* trail making task (seconds), *SC* symbol coding (number of boxes filled), *RTT* reaction time task (seconds), *IL-1b* interleukin 1 beta, *IL-1RA* interleukin 1 receptor antagonist *IL-6* interleukin 6, *IL-6Rsr* interleukin 6 soluble receptor, *IL-10* interleukin 10, *TNF* tumor necrosis factor, *sTNFR2* soluble tumor necrosis factor receptor 2, *MCP-1* monocyte chemoattractant protein-1.

*n = 26; **n = 28; †_*n*_ = 40; ‡_*n*_ = 42; §_*n*_ = 41.

### Psychomotor tasks

**Fig. 1:**
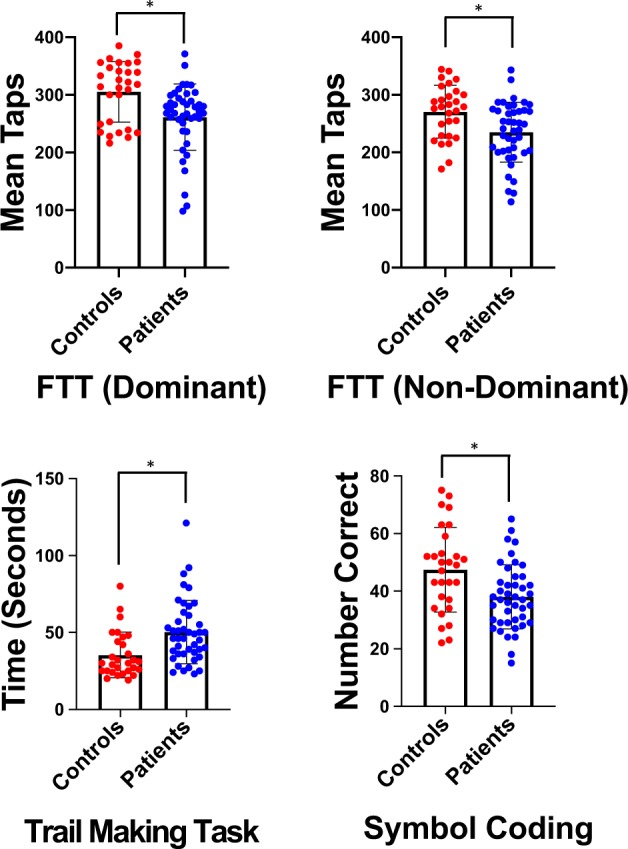
Mean task performance for psychomotor tasks. Mean performance on the finger tapping task (FTT) dominant, FTT non-dominant, trail making task, and symbol coding tasks for controls (red) and patients with schizophrenia (blue). *P* ≤ 0.001 for between group comparisons for all tasks.

Mean task performance for the psychomotor tasks is shown in Fig. 1. Patients were slower then controls on the psychomotor tasks, including the finger tapping test (FTT) for dominant hand (Wald chi-square = 11.085, *p* = 0.001) and non-dominant hand (Wald chi-square = 11.867, *p* = 0.001), the TMT (Wald chi-square = 15.980, *p* < 0.001), and the SC (Wald chi-square = 10.62, *p* = 0.001). The RTT was not different between groups (*p* > 0.05) and was excluded from other analyses. Estimated mean differences for all tasks can be found in Table 1.

### Immune markers

Generalized linear models found that patients had significantly higher concentrations of IL-1RA compared with controls (Wald chi-square = 5134, *p* = 0.023), whereas concentrations of TNF were higher in patients relative to controls at a trend level (Wald chi-square = 3.428, *p* = 0.064). Other markers including IL-1b, IL-6, IL-10, MCP-1, IL-6sr, and TNFR2 were not significantly different between patients and controls. Estimated mean differences for all immune markers can be found in Table 1.

### Effect of immune markers on psychomotor task performance

**Fig. 2:**
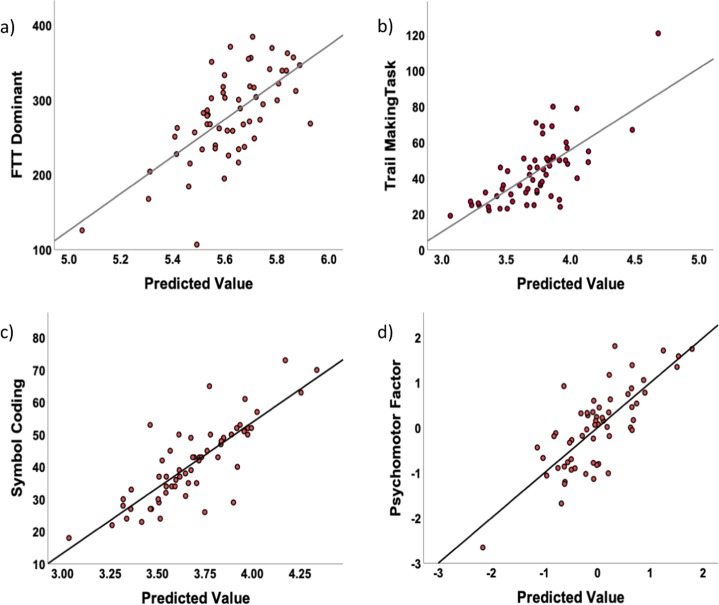
Actual task performance as predicted by statistical modeling including immune markers, diagnostic group, and covariates (predicted value). The correlation between the actual and predicted value for **a** finger tapping task (FTT) dominant was *r* = 0.666, *p* < 0.001, for **b** trail making task was *r* = 0.722, *p* < 0.001, for **c** symbol coding was *r* = 0.821, *p* < 0.001, and for **d** the psychomotor factor was *r* = 0.773, *p* < 0.001.

**Fig. 3:**
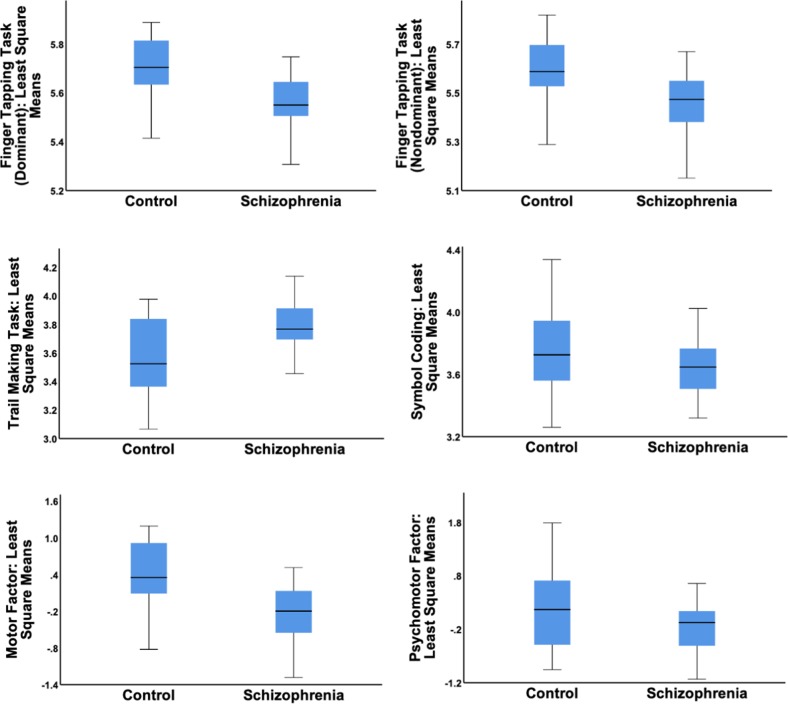
Least square means of predicted values for task performance in patients and controls. Least square means of the predicted values from the statistical models for the following tasks: finger tapping task (dominant hand and non-dominant hand), trail making task, and symbol coding task. In addition, the least square means of the predicted value from the statistical models for the motor and psychomotor factors from principle component analyses are included.

We used generalized linear models to test the impact of immune markers on psychomotor task performance, including the interaction of each marker by diagnosis on task performance. Table 2 lists the beta values and *p* values for each of these model results. Six of the available immune markers and their interactions with diagnosis were significantly associated with psychomotor task performance. IL-6 was significantly associated with slower performance on the SC and the IL-6 * diagnosis interaction met trend level significance for this task. Both TNF and the TNF * diagnosis interaction were associated with slower performance on three out of the four psychomotor tasks: FTT (dominant hand), TMT, and SC. The IL-6 soluble receptor (IL-6sr) was associated with slower performance on the finger tapping dominant task (dominant hand), and the IL-6sr * diagnosis interaction was significant for the FTT (dominant hand), FTT (non-dominant hand), and the SC. Both IL-1RA and the IL-1RA * diagnosis interaction were both significantly associated with SC. TNFR2, was significantly associated with SC, whereas the TNFR2 * diagnosis interaction was significant for SC and at trend level for FTT (dominant hand). Finally, the IL-10 and the IL-10 * diagnosis interaction was significant (or at trend level) for all four psychomotor tasks. Figure 2 shows actual by predicted plots, demonstrating the overall significance of the model testing the association between diagnostic group and immune markers with cognitive test performance after controlling for covariates. This suggests that our models appropriately reflect psychomotor task performance. Figure 3 shows least square mean values for the predicted values from the statistical models for patients and controls.

**Table 2:** Significant immune and demographic predictors of psychomotor tasks, including immune marker × diagnosis predictors (corrected *p* values based on Benjamini–Hochberg < 0.05).

Variable	Beta	Uncorrected *p* value	Corrected *p* value
FTT (dom)			
dx	−1.418	0.093	0.233
TNF	−0.070	0.002	0.015
dx * TNF	0.093	0.043	0.140
IL-10	0.252	0.051	0.150
dx * IL-10	−0.503	0.022	0.097
IL-6sr	−0.000007	0.017	0.083
dx * IL-6sr	0.00003	<0.001	<0.001
dx * TNFR2	0.000	0.051	0.150
FTT (non-dom)			
Sex	0.186	0.034	0.125
Age	−0.006	0.021	0.097
IL-10	0.301	0.042	0.140
dx * IL-10	−0.688	<0.001	<0.001
dx * IL-6sr	0.00002	0.005	0.034
TMT			
Sex	0.200	0.088	0.234
Age	0.013	0.025	0.103
TNF	0.112	0.026	0.103
dx * TNF	−0.186	0.006	0.038
IL-10	−0.604	0.001	0.008
dx * IL-10	1.372	<0.001	<0.001
TNFR2	0.000	0.091	0.234
SC			
dx	−1.831	0.027	0.103
Race	−0.167	0.015	0.083
Smoker	0.146	0.062	0.176
Age	−0.014	<0.001	<0.001
TNF	−0.056	0.045	0.141
dx * TNF	0.102	0.017	0.083
IL-6	−0.098	0.011	0.065
dx * IL-6	0.145	0.067	0.184
IL-10	0.311	<0.001	<0.001
dx * IL-10	−0.745	<0.001	<0.001
dx * IL-6sr	0.00002	0.001	0.008
IL-1ra	0.001	<0.001	<0.001
dx * IL-ra	−0.001	<0.001	<0.001
TNFR2	0.000	<0.001	<0.001
dx * TNFR2	0.0010	0.038	0.134

*FTT* (dom) finger tapping task (dominant hand; number of taps), *FTT* (non-dom) finger tapping task (non-dominant hand; number of taps); *TMT* trail making task (seconds), *SC* symbol coding (number of boxes filled), *IL-1RA* interleukin 1 receptor antagonist, *IL-6* interleukin 6, *IL-6sr* interleukin 6 soluble receptor, *IL-10* interleukin 10, *TNF* tumor necrosis factor, *TNFR2* tumor necrosis factor receptor 2.

Given the number of predictors included in the models, we performed Benjamini–Hochberg correction for multiple comparisons, which demonstrated that a number of the inflammatory markers and diagnosis * inflammatory marker predictors survived correction for multiple comparisons. The corrected *p* values from the Benjamini–Hochberg tests are included in Table 2.

### Principal components analysis

Two factors were extracted from the principal component analysis (PCA), namely a “motor factor” made up of the FTT for both the dominant and non-dominant hands, and a “psychomotor factor” that was made up of the TMT and the SC. The diagnosis * IL-6sr interaction (beta = 3.558, *p* = 0.002) was associated with the “motor factor”. For the psychomotor factor, sex (beta = −0.562, *p* = 0.029), age (beta = −0.037, *p* = 0.002), diagnosis * TNF interaction (beta = 0.967, *p* = 0.042), IL-10 (beta = 0.671, *p* = 0.022), diagnosis * IL-10 interaction (beta = −1.707, *p* < 0.001), IL-6sr (beta = 0.979, *p* = 0.053), IL-1RA (beta = 0.817, *p* = 0.004), TNFR2 (beta = −0.951, *p* < 0.001), and the diagnosis * TNFR2 interaction (beta = 0.968, *p* = 0.036) were significantly associated with slower performance. We additionally plotted psychomotor task performance with the predicted values from the above models to test whether the predicted values adequately predicted task performance while holding all other variables in the model constant (Figs. 2 and 3).

## Discussion

Patients with schizophrenia demonstrated significant slowing on a variety of psychomotor tasks, and this slower performance was associated with a number of peripheral immune markers in the patients. More specifically, there were interactive effects with diagnosis for TNF, IL-10, IL-6sr, and TNFR2 for the FTT (dominant), IL-10 and IL-6sr for FTT (non-dominant), TNF and IL-10 for TMT, and TNF, IL-10, IL-6sr, TNFR2, and IL-1RA for SC. Using principal component analysis, we found that the motor factor had a significant interactive effect between diagnosis and IL-6sr. The psychomotor factor, which comprised tasks involving both motor and additional cognitive processing, had significant interactive effects between diagnosis and TNF, IL-10, and TNFR2. Importantly, because the direction of the differences in inflammatory marker concentrations are different between groups for each individual marker, the sign of the beta values should be interpreted as absolute values to account for these differences in direction.

Only a few studies have demonstrated relationships between immune markers and psychomotor slowing in patients with schizophrenia. Kogan et al.^42^ found that TNF and IL-12p70 concentrations were negatively correlated with the speed of processing domain of the MCCB. This is in line with our finding that TNF predicted slower performance in patients with schizophrenia on both the TMT and SC tasks, which comprise two out of the three processing speed items from the MCCB, as well as our psychomotor component derived from the PCA.

Data from the FACE-SZ (FondaMental Academic Centers of Expertise for Schizophrenia) cohort^43^ showed relationships between worse performance on Trails A^44^ and increased CRP (>3 mg/L) but not the Digit Symbol Substitution Task^45^. However, using principle components analysis, the processing speed component (made up of TMT and SC) did not show a significant relationship with increased CRP. Similarly, in a study of 151 patients with schizophrenia, Frydecka et al.^46^ found significant associations between IL-6, but not CRP, and worse performance on both trails and SC. We found a diagnosis by IL-6 association at a trend level for SC, though the soluble IL-6 receptor was significant for all tasks except the TMT and was a significant predictor for the finger tapping component in the PCA analyses.

We did not include CRP in our analyses owing to its collinearity with many inflammatory cytokines. In addition, our previous study of psychomotor slowing and inflammation in patients with major depressive disorder did not show significant relationships between CRP and task performance, whereas individual inflammatory markers predicted slower performance^47^, similar to the results of this study. Furthermore, the wide array of immune markers we included in this study may give a more nuanced insight into which markers may be playing a role in psychomotor slowing in patients with schizophrenia, which in turn may represent putative drug targets in future studies of novel therapeutic agents to treat these cognitive deficits.

Regarding the specific immune markers, TNF, a pro-inflammatory cytokine, and IL-10, an anti-inflammatory cytokine, were associated with slowed task performance in patients with schizophrenia. Both cytokines have been shown to be elevated in patients with schizophrenia^25^. TNF has been associated with psychomotor slowing (see above) in patients with schizophrenia and also associates with negative symptom severity^32^ that in turn is associated with worse cognitive performance^48^. Decreased concentrations of IL-10 is associated with psychomotor slowing in patients with depression^47^ and in patients co-infected with HIV and hepatitis C^49^. The association between IL-10 and psychomotor slowing may act as a counter-regulatory mechanism in relation to the increased concentrations of pro-inflammatory markers such as TNF^50^. Moreover, concentrations of IL-10 are increased in meta-analyses of patients with schizophrenia, a finding that is believed to represent a counter-regulatory mechanism to balance the largely pro-inflammatory increases in patients with schizophrenia^25^.

Two inflammatory cytokine receptors, IL-6sr and TNFR2, were also significantly associated with psychomotor slowing in our subjects with schizophrenia. This is consistent with previous work in depression that showed increased concentrations of TNFR2 associated with worse performance on the FTT^47^, although TNFR2 was not associated with our finger tapping factor in the PCA analysis. IL-6sr binds IL-6, that in turn is associated with psychomotor slowing in previous studies of patients with schizophrenia (see above) as well as in our previous study in depression^47^. Moreover, IL-6 predicted slower key press reaction times on a Stroop task (in both congruent and incongruent conditions) in healthy volunteers given typhoid vaccination, a known inflammatory stimulus^35^. IL-6 is also associated with motor slowing in laboratory animals^51–53^.

These findings are consistent with previous work implicating a link between the basal ganglia and psychomotor slowing in patients with schizophrenia^12–15^ as well in other psychiatric and neurological disorders^54–57^. Relevant to our work, there is a growing literature implicating the basal ganglia and related circuitry as a target for inflammatory mediators^58^. For example, patients treated with the inflammatory cytokine interferon-alpha show increased glucose metabolism in the basal ganglia that is thought to be related to impaired dopamine-mediated inhibition of oscillatory burst activity in basal ganglia nuclei^59,60^. Similar findings have been reported in healthy individuals given typhoid vaccination who show decreased activation in the substantia nigra detected via fMRI, which in turn was associated with increased IL-6 and slowed performance on a reaction time task (RTT)^35^. Previous work in depression has also demonstrated that increased inflammation was associated with decreased functional connectivity between the dorsal striatum and the ventromedial prefrontal cortex, which in turn was associated with decreased performance on the FTT and the TMT^61^. Moreover, patients with depression and elevated CRP (≥ 5 m/g/L) who were treated with the TNF antagonist infliximab demonstrated significant improvement in psychomotor retardation compared with placebo^62^. It has yet to be demonstrated whether inflammation may target the basal ganglia or drive similar changes in connectivity between these regions in patients with schizophrenia.

There are several strengths of the current study: a hypothesis-driven approach, and an extensive panel of immune markers and psychomotor tasks that had not previously been studied together in patients with schizophrenia. In addition, the inclusion of a comparison group of healthy controls provided increased specificity for the relationship between immune markers and psychomotor performance in patients with schizophrenia. Regarding limitations, this study had a relatively small sample size that could have reduced power. It is unclear why there were not greater differences in immune marker concentrations between patients and controls, as previous studies and meta-analyses have shown differences between patients and controls. For example, we do not find a difference in IL-6 concentration, which has consistently been shown to be increased in patients with schizophrenia relative to controls. One reason for this, in part, is owing to the sample size limitations of this study. Another possible reason may be related to the fact that the patients included in this study are stable outpatients. Our previous meta-analysis demonstrated more robust group differences between patients and controls in patients with acute psychosis compared with those who were stable outpatients^25^. It should be noted that though there were nonsignificant differences in outpatients compared with controls, the significant IL-1RA finding and the trend finding for TNF, for example, are in the expected direction. Nonetheless, the lack of difference between groups may also be a strength in this study as it gives added weight to the relative contribution of immune markers to psychomotor slowing in patients with schizophrenia compared with controls. Given that the two groups had similar concentrations of inflammatory markers, this suggests that patients with schizophrenia may be uniquely sensitive to the effects of the immune system on the brain and behavior. Finally, these data are correlational in nature and as such, cannot prove cause and effect relationships. Future studies in animal models or interventional studies in humans are necessary to better understand the contribution of the immune system to psychomotor slowing in patients with schizophrenia. In summary, the results of this study provide evidence that peripheral inflammation in patients with schizophrenia, but not controls, may contribute to psychomotor slowing, a symptom of schizophrenia that has been consistently described. These data are also consistent with a growing literature, demonstrating that inflammation may target the basal ganglia to contribute to psychomotor deficits in a transdiagnostic fashion, with results that are similar to what has been shown in patients with depression. Future work using neuroimaging methods, for example, will be necessary to replicate the work from the mood disorders literature, demonstrating decreased functional connectivity with inflammation in areas of the brain that regulate motor activity. Interventional studies where inflammation is either induced or blocked will be important to demonstrate a causal relationship between inflammation and psychomotor slowing in patients with schizophrenia. Taken together, these data also indicate that psychomotor speed may be a relevant target in studies of the immune system and its impact on the brain in schizophrenia.

## Methods

### Description of study sample

Forty-three patients with schizophrenia or schizoaffective disorder and 29 healthy controls were recruited from the Atlanta Veterans Affairs Medical Center (Atlanta VAMC) and the local community. We did not perform one-to-one matching of demographic variables in the two diagnostic groups. Subjects were between 18 and 65 years old. Healthy controls had no history of major psychiatric disorder. Psychiatric diagnosis or lack thereof was confirmed by the Structured Clinical Interview for DSM-IV, Axis-I.^63^ In order to control for medical sources of inflammation or changes in psychomotor speed, subjects were excluded if they had a heart attack or heart failure within 6 months of screening, antibiotic use within the last 60 days, hospitalization during the last 60 days, any condition requiring steroids within the last 60 days, neurologic disease, head trauma, CNS infection, seizure disorder, intellectual disability, active substance abuse within 3 months of testing (as confirmed by SCID interview and urine toxicology on day of testing), HIV infection, autoimmune conditions, or clinically significant hearing or visual impairment. All subjects provided informed consent for the study approved by the Emory University Institutional Review Board and the Atlanta VAMC Research and Development Committee.

### Psychomotor speed tasks

Psychomotor performance was assessed by means of the following measures: (1) FTT, a motor test of the speed at which subjects are able to repeatedly press a key in a specified length of time^64^. Psychomotor speed in this test was measured as the number of taps the subject was able to complete in 60 seconds. The test was completed with both the dominant and non-dominant hand. (2) The TMT, also a subscale of the MCCB^5^, is a timed visuomotor task in which subjects draw a line to connect consecutively numbered circles placed irregularly on a sheet of paper. (3) Brief Assessment of Cognition in Schizophrenia SC, is a subscale of the MCCB^5^. It is a timed visuomotor test in which subjects use a key to write numbers that correspond to nonsense symbols. Processing speed is measured as the correct number of coded symbols in ninety seconds. (4) RTT^65^, is a visuomotor task consisting of the measurement of reaction time of key presses following simple visual stimuli.

### Immune assays

Blood samples for immune markers were obtained in chilled EDTA-coated tubes and spun at 2000 × *g* for 15 minutes at 4 °C, and plasma was collected and stored at −80 °C for later batched analysis of the inflammatory cytokines IL-6, IL-1 beta, and TNF and their soluble receptors, IL-6 soluble receptor (IL-6sr), IL-1 receptor antagonist (IL-1RA), and soluble TNF receptor 2 (TNFR2) as well as the anti-inflammatory cytokine IL-10 and the chemokine monocyte chemoattractant protein (MCP)-1, all of which have been found to be altered in patients with schizophrenia^25,26,31^. Concentrations of cytokines and the soluble receptors as well as MCP-1 were assessed in duplicate using high sensitivity multiplex bead-based assays (R&D Systems) and analyzed on a MAGPIX CCD imager (Luminex) as previous described^61,66^. All samples were run in the same batch and included high and low technical replicates as well as control plasma on each plate. Mean inter- and intra-assay coefficients of variation (CV) were reliably <10% (see Supplementary Table 1 for inter- and intra-assay CVs and method detection limits for each of the immune markers). No immune variables were below the limits of assay detection, although three subjects did not have enough sample volume for MCP-1, IL-6sr, IL-1RA, and TNFR2 assays.

### Statistical analyses

All statistical analysis was conducted in SPSS version 26.0 (IBM SPSS Statistics for Macintosh, Version 24.0. Armonk, NY: IBM Corporation). Normality assumptions were tested using standard tests (Shapiro–Wilks), and appropriate transformations were applied to non-normally distributed variables. Immune markers demonstrated non-normality even after transformations and hence generalized linear model with gamma-log link was used as it accommodates a wide variety of distributions (including gamma) and enables examination of several covariates within a single model. Gamma distribution with log link could not be used on data from principal component analysis (owing to negative loadings on some variables). The effect of group (patients vs. controls) differences upon psychomotor task performance and immune markers was first examined, followed by models to examine the association between immune markers and task performance across groups. Each individual psychomotor task was modeled as the response variable with diagnosis, race, sex, and smoking status (yes/no) as categorical and body mass index and age as continuous predictors. Both main effects and group * immune marker interactions were examined in accordance with the primary hypothesis of the study. Maximum likelihood (robust) estimation was used and parameter estimates were contrasted against null assumptions using log likelihood estimates. Post hoc margins were used to examine group-wise contrast of categorical factors and linear effects of continuous factors upon outcome variables of interest. Last, to examine the dimensionality in psychomotor changes, a PCA with varimax rotation was used to reduce the number of psychomotor tasks. A two-factor solution was extracted explaining 87.74% of the total variance. The two factors were then used as dependent variables in individual models that tested the effect of immune markers and diagnosis upon dimensions of psychomotor test performance.

In order to control for multiple comparisons, a Benjamini–Hochberg approach was employed^67^. This approach provides better control of type I error rates when conducting multiple hypothesis tests as compared with more conservative approaches^68,69^. We corrected for 22 individual predictors (diagnosis, demographic variables, inflammatory markers, and diagnosis * inflammatory marker interactions) across the four psychomotor tasks.

### Reporting summary

Further information on experimental design is available in the Nature Research Reporting Summary linked to this article.

## Supplementary information

Supplementary Materials

Reporting Summary

## Data Availability

The data that support the findings of this study are available from the corresponding author upon reasonable request.
