# Supplementary material for: Inflammatory markers are associated with psychomotor slowing in patients with schizophrenia compared to healthy controls
Source: NPJ Schizophr. 2020 Apr 1;6:8. doi: 10.1038/s41537-020-0098-4 (PMC7113262; doi:10.1038/s41537-020-0098-4)
Supplement: Supplementary file 1 — Supplementary Materials [file 41537_2020_98_MOESM1_ESM.docx]

**Supplementary Table 1.** Parameters for MAP assays for plasma cytokines and their soluble receptors

| Variable | Intra-assay CVs | Inter-assay CVs | MDL (pg/ml) |
| --- | --- | --- | --- |
| IL-1beta (pg/ml) | 3.85% | 2.26% | 0.16 |
| IL-1RA (ng/ml) | 2.78% | 1.92% | 8.19 |
| IL-6 (pg/ml) | 2.46% | 3.06% | 0.60 |
| sIL-6r (ng/ml) | 2.58% | 2.23% | 11.45 |
| IL-10 (pg/ml) | 8.54% | 3.21% | 0.09 |
| TNF (pg/ml) | 3.89% | 3.15% | 0.47 |
| sTNFR2 (ng/ml) | 6.22% | 2.77% | 10.70 |
| MCP-1 (pg/ml) | 5.42% | 2.23% | 16.24 |

CV: coefficients of variation; MAP: multi-analyte profiling; MDL: method detection limit; IL-1beta: interleukin 1 beta; IL-1RA: interleukin 1 receptor antagonist; IL-6: interleukin 6; sIL-6R: soluble interleukin 6 receptor; IL-10: interleukin 10; TNF: tumor necrosis factor; sTNFR2: soluble tumore necrosis factor receptor 2; MCP-1: monocyte chemoattractant protein 1

**Supplementary Table 2.** Calculation of effect sizes and power for significant predictors for each of the psychomotor tasks and factors created from principle component analysis.

| Psychomotor Task | Predictor | Degrees of Freedom | F Ratio | Probability | Partial Eta Squared | Adjusted Power |
| --- | --- | --- | --- | --- | --- | --- |
| FTT Dominant | Dx * IL-6sr | 1 | 13.843 | 0.0004 | 0.17784 | 0.9344 |
|  |  |  |  |  |  |  |
| FTT Nondominant | Dx | 1 | 8.7363 | 0.0044 | 0.12528 | 0.7662 |
|  | Dx * IL-10 | 1 | 5.7161 | 0.0199 | 0.08568 | 0.5535 |
|  |  |  |  |  |  |  |
| TMT | Dx * TNFR2 | 1 | 17.606 | <0.0001 | 0.22687 | 0.976 |
|  | Dx * IL-10 | 1 | 10.16 | 0.0023 | 0.14481 | 0.832 |
|  | Dx * IL-1b | 1 | 4.5218 | 0.0376 | 0.07008 | 0.4392 |
|  |  |  |  |  |  |  |
| SC | Age | 1 | 23.801 | <0.0001 | 0.28745 | 0.996 |
|  | Dx * TNFR2 | 1 | 18.056 | <0.0001 | 0.23432 | 0.9787 |
|  | Dx * IL-1RA | 1 | 8.8091 | 0.0043 | 0.12991 | 0.769 |
|  |  |  |  |  |  |  |
| Motor Factor | Dx * IL-6sr | 1 | 5.7295 | 0.0197 | 0.08336 | 0.5557 |
|  |  |  |  |  |  |  |
| Psychomotor Factor | Dx * TNFR2 | 1 | 19.205 | <0.0001 | 0.23945 | 0.9848 |
|  | Age | 1 | 15.456 | 0.0002 | 0.20215 | 0.9569 |
|  | Dx * IL-10 | 1 | 7.5912 | 0.0077 | 0.11067 | 0.698 |
|  | Race | 1 | 7.5111 | 0.008 | 0.10963 | 0.6927 |

FTT: Finger Tapping Task; Dx: Diagnosis; IL-6sr: Interleukin 6 soluble receptor; IL-1b: Interleukin 1 beta; IL-10: Interleukin 10; TMT: Trail Making Task; TNFR2: Tumor necrosis factor receptor 2; SC: symbol coding; IL-1RA: Interleukin 1 receptor antagonist
